# Clusterin negatively modulates mechanical stress-mediated ligamentum flavum hypertrophy through TGF-β1 signaling

**DOI:** 10.1038/s12276-022-00849-2

**Published:** 2022-09-21

**Authors:** Chunlei Liu, Peng Li, Xiang Ao, Zhengnan Lian, Jie Liu, Chenglong Li, Minjun Huang, Liang Wang, Zhongmin Zhang

**Affiliations:** 1 Division of Spine Surgery, Department of Orthopedics, Nanfang Hospital, Southern Medical University, Guangdong Province Guangzhou, China; 2 Division of Spine Surgery, Department of Orthopedics, The Sixth Affiliated Hospital of Guangzhou Medical University, Qingyuan People’s Hospital, Guangdong Province Qingyuan, China; 3 Department of Orthopedics, The Third Affiliated Hospital, Southern Medical University, Academy of Orthopedics, Guangdong Province Guangzhou, China

**Keywords:** Stress signalling, Translational research, Experimental models of disease, Proteomic analysis, Antibody therapy

## Abstract

Ligamentum flavum hypertrophy (LFH) is a major cause of lumbar spinal canal stenosis (LSCS). The pathomechanisms for LFH have not been fully elucidated. Isobaric tags for relative and absolute quantitation (iTRAQ) technology, proteomics assessments of human ligamentum flavum (LF), and successive assays were performed to explore the effect of clusterin (CLU) upregulation on LFH pathogenesis. LFH samples exhibited higher cell positive rates of the CLU, TGF-β1, α-SMA, ALK5 and p-SMAD3 proteins than non-LFH samples. Mechanical stress and TGF-β1 initiated CLU expression in LF cells. Notably, CLU inhibited the expression of mechanical stress-stimulated and TGF-β1-stimulated COL1A2 and α-SMA. Mechanistic studies showed that CLU inhibited mechanical stress-stimulated and TGF-β1-induced SMAD3 activities through suppression of the phosphorylation of SMAD3 and by inhibiting its nuclear translocation by competitively binding to ALK5. PRKD3 stabilized CLU protein by inhibiting lysosomal distribution and degradation of CLU. CLU attenuated mechanical stress-induced LFH in vivo. In summary, the findings showed that CLU attenuates mechanical stress-induced LFH by modulating the TGF-β1 pathways in vitro and in vivo. These findings imply that CLU is induced by mechanical stress and TGF-β1 and inhibits LF fibrotic responses via negative feedback regulation of the TGF-β1 pathway. These findings indicate that CLU is a potential treatment target for LFH.

## Lower back pain: clusterin control for tissue repair

The protein clusterin regulates the body’s response to lower back pain induced by mechanical stress and could be a target for treatments. Lower back pain is common and is exacerbated by our upright stance. A major cause of the pain is excessive cell growth (hypertrophy) in the ligaments between vertebrae. This growth narrows the spinal canal and compresses nerves. Using a unique mouse model bred to walk upright, Zhongmin Zhang and Liang Wang at Southern Medical University in Guangzhou, China, and co-workers showed that clusterin, a protein involved in regulation of cell survival, can reduce the hypertrophy caused by mechanical stresses, and could be used in back pain treatments. Clusterin regulates the activity of the growth factor TGF-β1, which plays a role in synthesizing new tissues after injury, but can spur excessive growth.

## Introduction

Low back pain (LBP) is a major chronic disease worldwide and places a heavy economic burden on patients and society. Approximately 70% of the global population experiences LBP at least once in their lifetime^1^. Clinically, lumbar spinal canal stenosis (LSCS) is a major cause of LBP. LSCS manifests as hypertrophy of the elements surrounding the spinal canal, including the ligamentum flavum (LF), joint osteophytes, intervertebral disc, and joint capsule, which narrows the available space for the dural sac as well as its neuronal contents. Among them, ligamentum flavum hypertrophy (LFH) is highly correlated with LSCS pathogenesis^2,3^. LFH tissue is characterized by altered arrangements of fibers in patients with LSCS. These changes alter extracellular matrix (ECM) remodeling, leading to ligament fibrosis and an increase in the thickness of LF tissue^4^.

LFH, a multifactorial disease, is related to mechanical stress, age, lumbar disk herniation and obesity^5^. In our previous study^6^, a novel bipedal standing mouse model was established. Compared with quadruped mice, bipedal standing mice induced larger mechanical stress in the lumbar spine. As a result, the LF area in bipedal standing mice subjected to mechanical stress for 10 weeks was larger than the LF area in quadruped mice and even larger than that in 18-month-old mice. Moreover, lumbar disc herniation or obesity induces mechanical stress on the LF, resulting in LFH. Clinically, spinal segmental instability caused by disk and facet joint degeneration leading to abnormal mechanical stress has been reported in patients with LSCS. In addition, increasing instability plays a vital role in the pathogenesis of LFH^7,8^. These findings indicate that mechanical stress is an important factor for the pathogenesis of LFH.

In LF tissues, mechanical stress can lead to microinjury. Recurrent microinjuries induce chronic inflammation and tissue fibrosis^9^. As an important inflammatory cytokine, transforming growth factor-beta 1 (TGF-β1) is induced by mechanical stress, which subsequently mediates collagen fiber synthesis and the loss of elastin fibers, ultimately causing LFH^10^.

ECM deposition in mechanical stress-mediated fibrosis in several tissues has been shown to be regulated by TGF-β1. Synthesis of the TGF-β1 precursor occurs intracellularly, from where it is secreted into the ECM as an inactive latent complex to bind the cell surface receptor Activin receptor-like kinase 5 (ALK5) to elicit downstream signaling through the canonical SMAD3 pathway. The SMAD3 pathway mainly mediates fibrotic responses, and TGF-β1 stimulates SMAD3 and promotes phosphorylation of SMAD2 as well as nuclear translocation to activate gene transcription in relation to SMAD4.

TGF-β1-induced ECM synthesis plays a key role in LF scar repair; however, excessive deposition of the ECM leads to fibrosis and LFH. Therefore, it is imperative to identify mechanisms that promote appropriate resolution of TGF-β1 signaling. Previous studies have reported that clusterin (CLU) is a negative feedback regulator of TGF-β1 signaling and ECM deposition in renal fibrosis^11^ and hepatic fibrosis^12^. CLU is present in many human tissues, with the secreted protein being present in all body fluids^13^. Two isoforms are encoded by the CLU gene: a truncated nuclear form and a conventional omnipresent secretory heterodimeric disulfide-linked glycoprotein^14^. CLU is highly expressed in patients with diabetes^15^, atherosclerosis^16^, and tumorigenesis^17^. The role of CLU in LFH has not been fully explored. Therefore, this study aimed to investigate the function of CLU in LFH and ECM metabolism. Furthermore, the regulation of TGF-β1 pathways by CLU in LF tissue was explored.

## Materials and methods

### LF tissue samples and blood samples

A total of 44 LF samples (LFH: non-LFH = 22:22) and 42 blood samples were obtained from patients who had undergone surgical therapy at the Third Affiliated Hospital of Southern Medical University. The quantity demands of LF samples for each experiment were as follows: proteomics experiment (LFH: non-LFH = 3:3), RT‒PCR experiment (5:5), Western blot assays (8:4), histological staining assays (6:6) and cell assays (non-LFH = 4). LF thickness was determined by MRI examination. LF samples were obtained from the L4/5 central portion of LF. Prior to surgery, patients were required to provide informed consent.

### Isobaric tags for relative and absolute quantitation of LF samples

LF samples were obtained from three pairs of patients diagnosed with LFH and non-LFH patients. Protein extraction and iTRAQ proteomics were conducted following previously described methods^6^. LF tissues were homogenized in liquid nitrogen, after which they were lysed, sonicated, centrifuged and digested. iTRAQ (Applied Biosystems, USA) labeling was conducted based on the manufacturer’s protocols. High-pH fractionation was conducted using the Ultimate 3000 HPLC system (Dionex, USA).

### Proteomic and bioinformatics analyses

Protein Pilot Software 5.0 (AB SCIEX, USA) was used to analyze peptide data. Proteins with ≥1 unique peptide and unused value >2 were selected for subsequent assessments. Log2 FC (fold change) > 2.0, *p* < 0.01, and CV < 0.5 were used to establish significantly differentially expressed proteins. Kyoto Encyclopedia of Genes and Genomes (KEGG) and Gene Ontology (GO) enrichment analyses were conducted using the DAVID (http://david.abcc.ncifcrf.gov) tool to determine the functions of selected proteins. Protein-protein interaction network (PPI) analysis was conducted using the STRING tool (www.string-db.org).

### Cell cultures and treatment

LF samples were acquired from patients who had been subjected to spinal surgery. LF samples were minced and digested using type I collagenase (0.2%, Gibco, USA) at 37 °C for 1 h. After digestion, LF tissues were washed with DMEM (Gibco) and transferred to one-well plates (Corning-Costar, USA) containing DMEM with 20% FBS (Gibco), 100 mg/ml streptomycin and 100 U/ml penicillin (Gibco). The samples were then incubated at 37 °C in a 5% CO_2_ humid environment. The culture medium was replaced after every 3 days. The migration of LF cells out of LF tissues started after ~10–16 days, and the cells formed a monolayer. Cells at passage <P5 were obtained for subsequent experiments.

ALK5 and protein kinase D3 (PRKD3)-targeting small interfering RNAs (siRNAs) were obtained from Guangzhou TSINGKE Biotech. Primer sequences are presented in Supplementary Table 1. ALK5-overexpressing and PRKD3-overexpressing pcDNA3.1 plasmids were synthesized by Guangzhou Laisai Biotech. Cells were transfected with siRNAs and plasmids using Lipo3000^TM^ Transfection Reagent (Invitrogen, USA) at the following final concentrations: 50 nM siALK5, 50 nM siPRKD3, 2 µg/ml ALK5 plasmid and 2 µg/ml PRKD3 plasmid as described by the manufacturers. After 48 h of transfection, the cells were subjected to different treatments.

Cells were treated with TGF‐β1 (5 ng/ml, SinoBiological, China), CLU (0.1–1 µg/ml, NovoProtein, China), MG132 (10 µM, MedChemExpress, China), CQ (10 µM, MedChemExpress), or CRT0066101 (1 µM, MedChemExpress) before harvesting.

### Application of mechanical stretch on LF cells

LF cells were cultured in six-well Bioflex plates (BioFlex, USA), incubated to 80–90% confluence and serum-starved for 24 h. LF cells were subjected to cyclic stretching with 10 s of 20% elongation and 10 s of relaxation for 8 h or 16 h using an FX5000 Tension (Flexcell International, USA) stretching device^10^. The control group was subjected to similar conditions but without mechanical stretching. The culture solution obtained after the mechanical stretch application procedure was used for ELISAs, and cells were used for protein/RNA extraction or immunofluorescence staining.

### Cell viability analysis

A Cell Counting Kit-8 (CCK8, Beyotime, China) assay was performed to evaluate cell viability. LF cells (5×10^3^/well) were seeded into 96-well plates. Cells were cultured to 80–90% confluence and incubated with varying CLU concentrations for 24 h. Each well was supplemented with 10 µl of CCK8 followed by incubation for 2 h. A microplate reader (BioTek, USA) was used to read absorbance at 450 nm. Data for each group were normalized to that of the control group.

### Western blotting

LF tissues were ground using liquid nitrogen, and cells were washed using PBS. Suspended cells (in RIPA lysis buffer) were lysed on ice. An 8% separation gel and a 5% stacking gel were used for electrophoresis. Through the wet transfer method, separated proteins were transferred to nitrocellulose membranes. Membrane blocking was achieved using 5% milk for 1 h, after which they were incubated with primary antibodies against TGF-β1 (1:1000, ABclonal, China), COL1A2 (1:1000, ABclonal), a-SMA (1:1000, ABclonal), CLU (1:1000, ABclonal), ALK5 (1:1000, ABclonal), SMAD3 (1:2000, Abcam, UN), p-SMAD3 (1:2000, Abcam) and GAPDH (1:1000, ABclonal) at 4 °C overnight. Visualization was conducted using enhanced chemiluminescence (Affinity, USA), while a Quantity One system (Syngene, UN) was used for quantitative analysis of proteins.

### RT-PCR

Total RNA extraction from LF tissues or cells was performed using TRIzol (Thermo, USA) reagent as instructed by the manufacturer. PrimeScript RT Master Mix (TaKaRa, Japan) was used to synthesize cDNA from extracted RNA. mRNA levels were determined by the SYBR Green I incorporation method and a real-time PCR system (Syngene, UN). Fold changes in relative gene levels were calculated by the 2^−ΔΔCt^ method. Primers were obtained from Shanghai Biological Engineering (Supplementary Table 2).

### ELISA

Total levels of TGF-β1, CLU and COL1A2 in culture medium collected after mechanical stress experiments were determined using an ELISA kit (ABclonal). Blood serum clusterin concentrations were determined using Human CLU and PRKD3 ELISA kits (Jingmei, China) as instructed by the manufacturers.

### Coimmunoprecipitation

Coimmunoprecipitation experiments were conducted as previously reported^18^. Lysis buffer (Beyotime) was used to lyse LF. Cell lysates were incubated with anti-antibody at 4 °C for 2 h, and then, protein A/G Agars purchased from Thermo Scientific were added and incubated for another 2 h. Protein A/G Agars were washed 5 times using lysis buffer before elution using SDS protein loading buffer at 95 °C. The protein was further used for western blot analysis.

### Histological analyses

LF samples from human or mouse tissues were fixed, decalcified, and embedded into paraffin blocks. Samples were sliced into 4 μm thick sections. Section staining was performed using H&E staining kits (Sigma, United States), Masson’s trichrome (MT) staining kits (Absin, China) or elastic van Gieson (EVG) staining kits (Baso, China) as instructed by the manufacturers. The images magnified 6100 (NIH, USA) tool was used for quantitation of LF and analysis of the ratio of collagen fibers to elastic fibers^6,19^.

### Immunohistochemistry and immunofluorescence analysis

Antigen removal from tissue sections was performed using citrate buffer at 60 °C for 16 h. Immunohistochemistry (IHC) sections were treated for 15 min with hydrogen peroxide and blocked for 1 h using 1% goat serum. LF cells for immunofluorescence (IF) analysis were fixed in paraformaldehyde (4%), incubated in the presence of Triton-100 (0.5%) and blocked using bovine serum albumin (BSA, 5%) at room temperature for 2 h. Overnight incubation of the sections and cells at 4 °C was performed in the presence of primary antibodies against CLU (1:100, ABclonal; 1:100, Santa Cruz, USA), TGF-β1 (1:100, ABclonal), α-SMA (1:100, ABclonal), p-SMAD3 (1:100, Abcam), COL1A2 (1:100, ABclonal), vimentin (1:200, Santa Cruz), ALK5 (1:100, ABclonal), PRKD3 (1:100, Proteintech, China), and LAMP1 (1:50, Santa Cruz). Samples were washed, after which they were incubated with matched secondary antibody for 1 h. Hematoxylin (Beyotime) and DAB (Beyotime) stains were used for IHC, while DAPI stain was used to stain the nuclei during IF analysis.

### Animal assays

Male C57BL/6 mice (8 weeks old) were procured from the Experimental Animal Center of Southern Medical University. Mouse LFH models were established as previously described by taking advantage of the hydrophobia of mice to induce them to adopt a bipedal standing posture for 6 h a day with an interval of 2 h of free activity^6^. Animals were randomized into the control, bipedal standing, saline + bipedal standing and CLU + bipedal standing groups (*n* = 6). All treatments were conducted under anesthesia, which had been induced by intraperitoneal administration of chloral hydrate. C57BL/6 mice in the CLU + bipedal standing and saline + bipedal standing groups received 16 tail intravenous doses of CLU (1 mg/kg) or saline for 8 weeks (2 doses/week)^20^. After the mice had been euthanized, intact L5/6 vertebrae were resected after completion of treatment. HE, MT, EVG and immunohistochemistry analyses were performed on the obtained samples.

### Statistical analysis

SPSS 25.0 software (SPSS, USA) was used for data analyses. Graphs were generated using GraphPad Prism 7.1.0 Software (GraphPad, CA). Comparisons of means between groups were conducted by Student’s *t*-test, while one-way ANOVA was used for among-group comparisons. Data are shown as the mean ± S.D. The threshold for significance was *P* ≤ 0.05.

## Results

### Proteomic data analysis and bioinformatics analysis of LFH

**Fig. 1:**
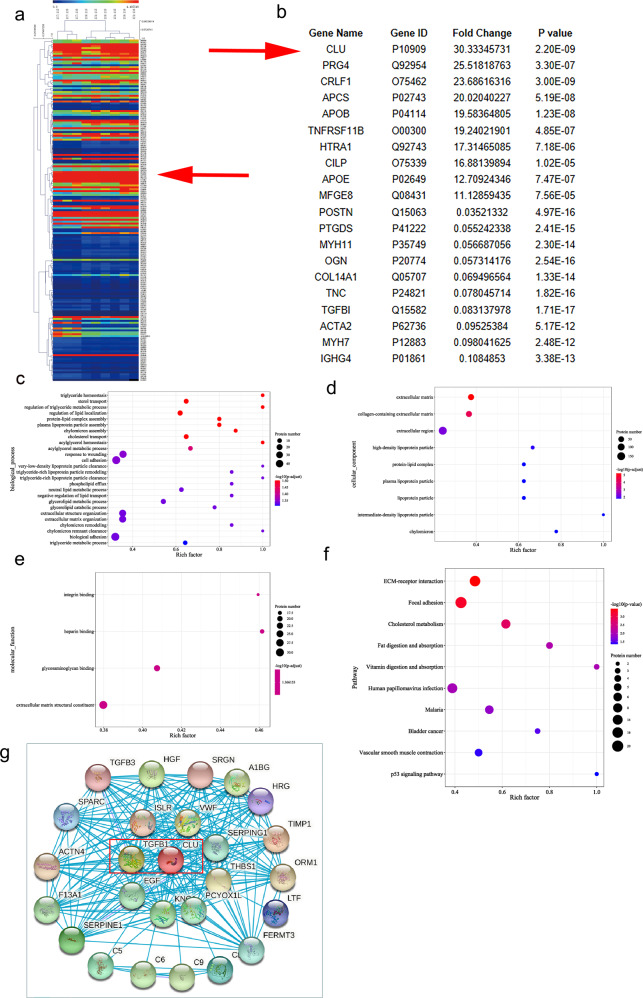
Proteomic profile of LF as determined by mass spectrometry analysis and bioinformatic analysis. **a** A cluster heatmap showing differentially expressed proteins with over 2-fold changes between LFH and non-LFH samples. Red denotes elevated levels, while green denotes decreased levels. Red arrow denotes CLU. **b** Top 10 up- and top 10 downregulated proteins. Levels of CLU protein in LFH were elevated by >30-fold compared with the control. GO and KEGG pathway analysis. **c**–**e** GO terms for differentially expressed proteins (*p* < 0.05). The color shows the enrichment scores (−lg(*p*-value)), and the number of proteins tested is proportional to the marker area of the bubble of the significantly enriched GO terms. **c** Biological process (BP) GO terms. **d** Cellular component (CC) GO terms. **e** Molecular function (MF) GO terms. **f** Pathways associated with differentially expressed proteins in LFH (*p* < 0.05). **g** Protein‒protein interaction (PPI) analysis using the STRING tool showing functional association networks of differentially expressed proteins. The associations between nodes are connected with solid lines. Red marked rectangle shows CLU-TGF-β1 interactions.

Hierarchical clustering of all expressed proteins was used to explore differential protein expression profiles in LFH and non-LFH tissue samples (three LFH and three non-LFH samples). In this study, 2328 proteins were identified and quantified. Notably, 179 differentially expressed proteins (DEPs) with fold changes > 2.0 were detected. Among the 179 differentially expressed proteins, 84 proteins had elevated levels, while 95 proteins had decreased levels. Cluster heatmaps were generated for the DEPs with more than a 2-fold increase or at least a 0.5-fold decrease and a *P-*value < 0.01 (Fig. 1a). The top 10 upregulated proteins and top ten downregulated proteins were then selected for subsequent analysis (Fig. 1b). The expression of CLU was upregulated by >30-fold in the LFH group compared with the non-LFH group.

The functional roles of novel proteins can be indirectly predicted by exploring the functions of selected proteins. GO and KEGG pathway analyses were performed on differentially expressed proteins to explore underlying biological functions and associated pathways (*P* < 0.05). GO analysis indicated that the DEPs were enriched in 26 biological processes (Fig. 1c). Differentially expressed proteins were enriched in 9 cellular components (Fig. 1d). Moreover, the proteins were enriched in 4 molecular functions (Fig. 1e). The most enriched protein was related to fibrosis-associated biological behaviors. KEGG analysis showed that differentially expressed proteins were implicated in pathways associated with fibrosis, such as the ECM-receptor interaction pathway, focal adhesion pathway and cholesterol metabolism pathway (Fig. 1f). PPI analysis using the STRING tool showed that TGF-β1 was associated with CLU (Fig. 1g).

### CLU and fibrosis-related expression were upregulated in LFH

**Fig. 2:**
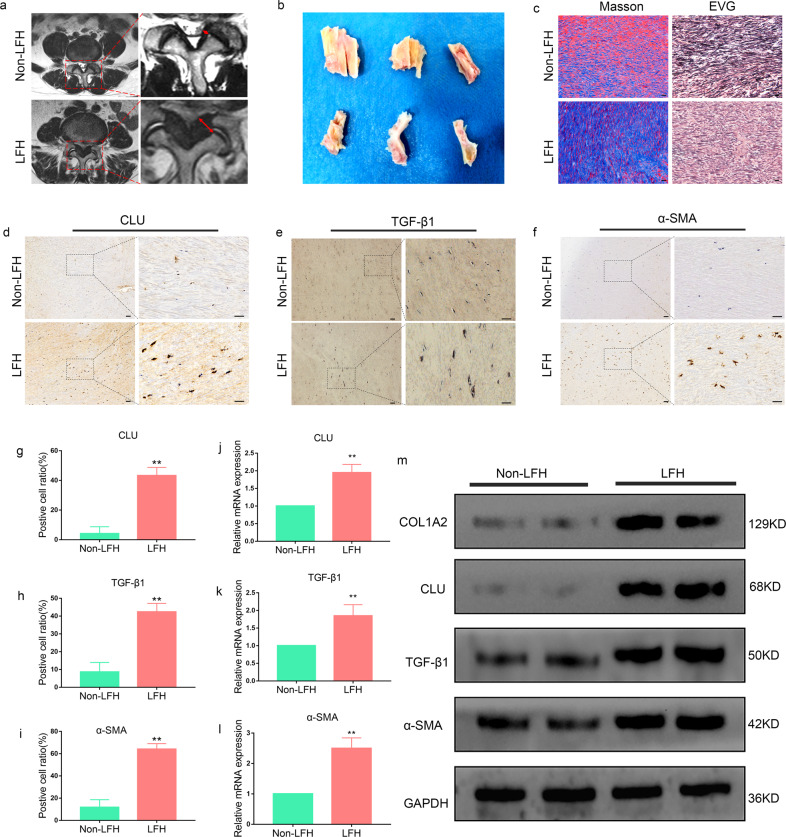
CLU and fibrosis-related expression are upregulated in LFH. **a** Preoperative determination of the LF thickness by MRI. The red dotted area was chosen for analyses. The red arrow denotes LF thickness at facet joint levels. **b** Representative LF samples obtained from non-LFH patients and LFH patients during spinal surgery. **c** Representative images of Masson’s trichrome and EVG staining of the LF samples from the LFH group and non-LFH group. Masson’s trichrome staining (elastic fibers are stained crimson, while collagen fibers are stained blue); EVG staining (elastic fibers are stained black, while collagen fibers are stained pink). Scale bar indicates 50 μm. **d**–**f** Immunohistochemical staining of CLU, TGF-β1 and α-SMA in LF tissues from the two groups (*n* = 6). The scale bar indicates 100 μm. **g**–**i** Quantitative analysis of the positive cell ratio for CLU, TGF-β1 and α-SMA. Error bars: S.D. **P < 0.01. **j**–**l** Validation of gene expression levels by RT‒PCR. The mRNA expression levels of CLU, TGF-β1 and α-SMA were markedly higher in the LFH group (*n* = 6) than in the non-LFH group (*n* = 6). Relative mRNA levels were normalized to GAPDH via the comparative Ct method. Data are expressed as the mean ± S.D. **P < 0.01. **m** Western blot analysis of CLU, TGF-β1, α-SMA, and COL1A2 protein expression in LF tissues from the two groups (*n* = 6). GAPDH was the loading control.

The results from proteomics analysis were validated by determining CLU expression levels by IHC, RT‒PCR and western blot analysis. The LF thickness for each patient was determined through MRI analysis (Fig. 2a). The mean LF thickness in the LFH group (5.91 ± 0.20 mm) was markedly higher than that in the non-LFH group (3.05 ± 0.09 mm) (Supplementary Table 3). Moreover, the analysis showed a significant difference in age between the two groups. Representative LF tissues were obtained during surgery (Fig. 2b). MT- and EVG-stained sections showed an increase in collagen fibers and a marked loss of elastic fibers in the LFH group; however, no significant changes were observed in the non-LFH group (Fig. 2c). IHC analysis was then performed to verify the results. The findings showed that the positive cell ratios for CLU, TGF-β1, and α-SMA were increased in all LFH samples relative to non-LFH samples (Fig. 2d–i). In addition, upregulation of CLU and markers of fibrosis was observed at the protein level in the LFH group through western blot analysis (Fig. 2m). Furthermore, RT‒PCR analysis showed a significant increase in CLU, TGF-β1 and α-SMA mRNA levels in the LFH group compared to the non-LFH group (Fig. 2j–l).

### Expression levels of CLU and TGF-β1 were induced by mechanical stress in LF cells

**Fig. 3:**
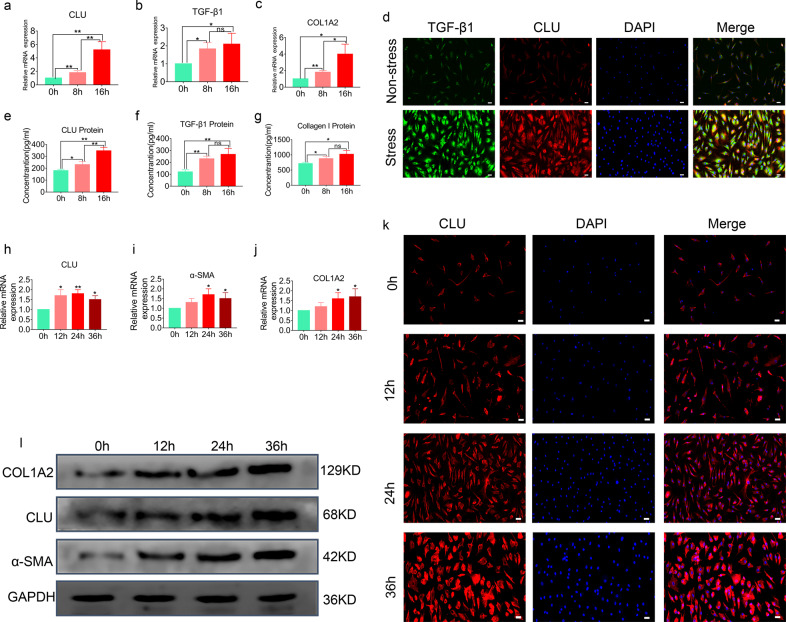
Mechanical stress and TGF-β1 induce the expression of CLU in LF cells. **a**–**c** Quantitative RT‒PCR analysis showing the gene expression of TGF-β1, CLU and COL1A2 in LF cells after treatment for 8 h or 16 h under mechanical stress. Relative mRNA expression levels for nontreated cells were set as 1. Data are expressed as the mean ± S.D. *P < 0.05, **P < 0.01. **d** The nonstress group showed fibroblast‐like morphology with weak staining of TGF-β1 and CLU. The stress group showed a higher intensity of TGF-β1 and CLU staining after applying mechanical stress for 16 h. TGF-β1 (green), CLU (red), DAPI (blue); the scale bar indicates 50 μm. **e**–**g** ELISAs of TGF-β1, CLU and collagen I protein levels in culture medium after subjecting samples to mechanical stress for 8 h or 16 h. Data are shown as the mean ± S.D. *P < 0.05, **P < 0.01. **h**–**j** Quantitative RT‒PCR analysis showing the gene expression levels of CLU, α-SMA and COL1A2 in LF cells after TGF-β1 (5 ng/mL) treatment for the indicated duration. Relative mRNA expression levels for nontreated cells were set as 1. Data are expressed as the mean ± S.D. *P < 0.05, **P < 0.01. **k** Fibroblast‐like morphology with staining of CLU after treatment with 5 ng/mL TGF-β1 for the indicated duration showed an increase in staining intensity in a time-dependent manner. CLU (red), DAPI (blue); the scale bar indicates 50 μm. **l** Western blot analysis showing the protein levels of CLU, α-SMA and COL1A2 in LF cells after TGF-β1 (5 ng/mL) treatment for the indicated duration for the four groups. The expression levels of CLU, α-SMA and COL1A2 increased in a time-dependent manner.

A mechanical stress cyclic stretch model using LF cells was used to investigate the importance of CLU in mechanical stress-mediated cellular fibrotic changes. After 8 h or 16 h of mechanical stress exposure, gene expression analysis was conducted using RT‒PCR. A time-dependent increase in CLU mRNA levels was observed after subjecting cells to mechanical stress (Fig. 3a). Furthermore, under mechanical stress, the extracellular CLU protein level was time-dependently increased (Fig. 3e). Moreover, TGF-β1 and COL1A2 mRNA and protein levels increased under mechanical stress in LF cells (Fig. 3b, c, f, g). Notably, TGF-β1 and CLU staining was weak in the nonstress group, indicating low expression levels. LF cells showed stronger staining of TGF-β1 and CLU after applying mechanical stress for 16 h (Fig. 3d).

### CLU expression was induced by TGF-β1 in LF cells

We found that CLU and fibrosis-related expression of LF cell markers were upregulated upon TGF-β1 treatment. The results showed that TGF-β1 induced a time-dependent increase in CLU mRNA levels (Fig. 3h). Similarly, TGF-β1 increased CLU protein expression levels (Fig. 3l). Moreover, the LF cell fibrotic markers COL1A2 and α-SMA were upregulated after TGF-β1 treatment (Fig. 3i, j, l). However, the mRNA and protein levels of CLU increased at a higher rate than those of α-SMA and COL1A2 upon TGF-β1 treatment. The intensity of CLU staining increased after TGF-β1 treatment in a time-dependent manner (Fig. 3k).

### CLU inhibited TGF-β1-induced SMAD3 signaling

**Fig. 4:**
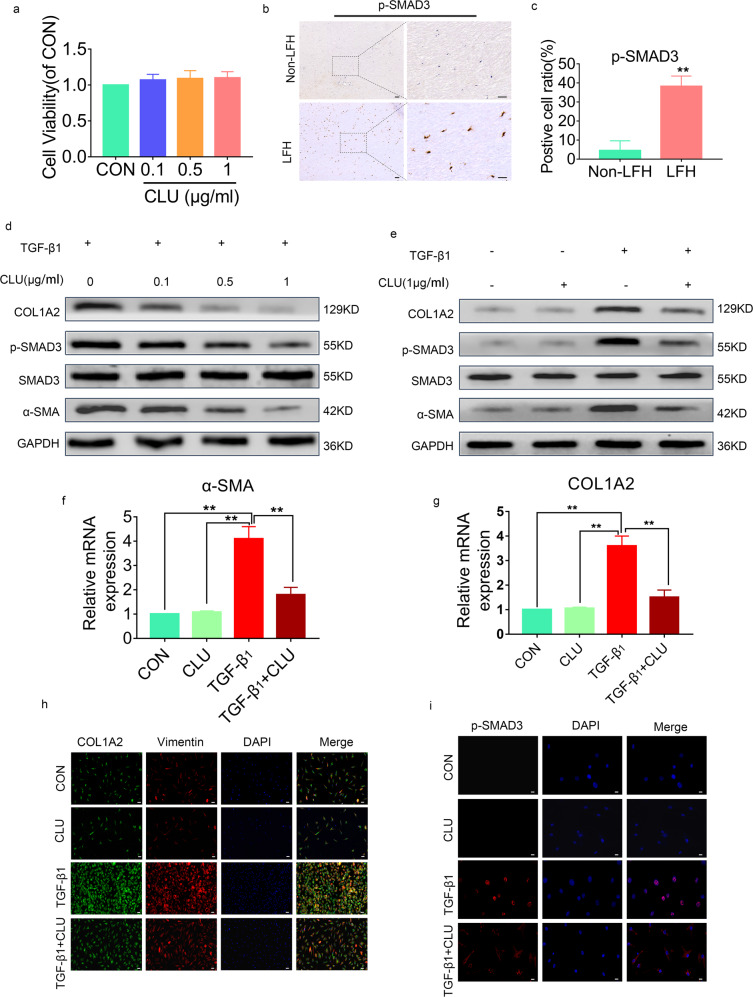
CLU inhibits TGF-β1-induced SMAD3 signaling. **a** Cell viabilities were evaluated by the CCK8 assay (*n* = 3). Data are shown as the mean ± SD. **b** Immunohistochemical staining of p-SMAD3 in LF tissues from the two groups (*n* = 6). The scale bar indicates 100 μm. **c** Positive cell ratio for p-SMAD3. Data are expressed as the mean ± S.D. **P < 0.01. **d** Western blot analysis showing the protein expression levels of SMAD3 signaling in LF cells after treatment with 5 ng/mL TGF-β1 and different concentrations of CLU for 24 h. **e** SMAD3 signaling protein levels in LF cells after different treatments for 24 h. **f**, **g** Quantitative RT‒PCR analysis showing the gene expression levels of α-SMA and COL1A2 in LF cells from different treatment groups. Relative mRNA expression levels from control group cells were set as 1. Data are shown as the mean ± S.D. *P < 0.05, **P < 0.01. **h** Immunofluorescence staining of COL1A2 and Vimentin under different treatments for 24 h. COL1A2 (green), Vimentin (red), DAPI (blue); the scale bar indicates 50 μm. **i** Effects of CLU on p-SMAD3 nuclear translocation in LF cells. The cells had different treatments for 24 h. p-SMAD3 (red), DAPI (blue), the scale bar indicates 20 μm.

Further analysis was conducted to explore the ability of CLU to inhibit TGF-β1-stimulated profibrotic target gene expression and protein expression in cultured LF cells by SMAD3 signaling. A CCK8 assay was performed to determine the potential toxic effect of CLU on cell proliferation. At 48 h post-treatment with CLU, there were no marked differences in cell proliferation among the groups. This finding implied that stimulation with CLU, even at 1 µg/mL, did not exert toxic effects on LF cell growth (Fig. 4a).

IHC was performed to verify that the ratio of p-SMAD3-positive cells was increased in LFH samples (Fig. 4b, c). Western blot assays revealed that administration of CLU to LF cells suppressed TGF-β1-stimulated p-SMAD3, α-SMA, and COL1A2 protein expression in a dose-dependent manner (Fig. 4d, e). Moreover, RT‒PCR analysis showed that administration of CLU in LF cells inhibited TGF-β1-stimulated α-SMA and COL1A2 expression at the mRNA level (Fig. 4f, g). Furthermore, immunofluorescence staining analysis showed that CLU inhibited the protein expression of TGF-β1-stimulated vimentin and COL1A2. However, CLU had no marked effects on basal mRNA or protein expression of α-SMA and COL1A2 before TGF-β1 treatment (Fig. 4h).

SMAD3 phosphorylation (p-SMAD3) and its successive nuclear translocation are important in TGF-β1 signaling. Thus, the effects of CLU on p-SMAD3 and its nuclear translocation were explored. The findings showed that p-SMAD3 levels increased in the nucleus upon TGF-β1 stimulation (Fig. 4i). However, increased p-SMAD3 levels in LF cells after TGF-β1 treatment were inhibited by CLU administration. These results imply that extrinsic CLU blocked p-SMAD3 translocation into the nucleus. In summary, these findings indicate that CLU negatively affects TGF-β1-induced transcription through suppression of p-SMAD3 as well as its nuclear translocation.

### CLU inhibited TGF-β1-induced SMAD3 signaling by binding to ALK5

**Fig. 5:**
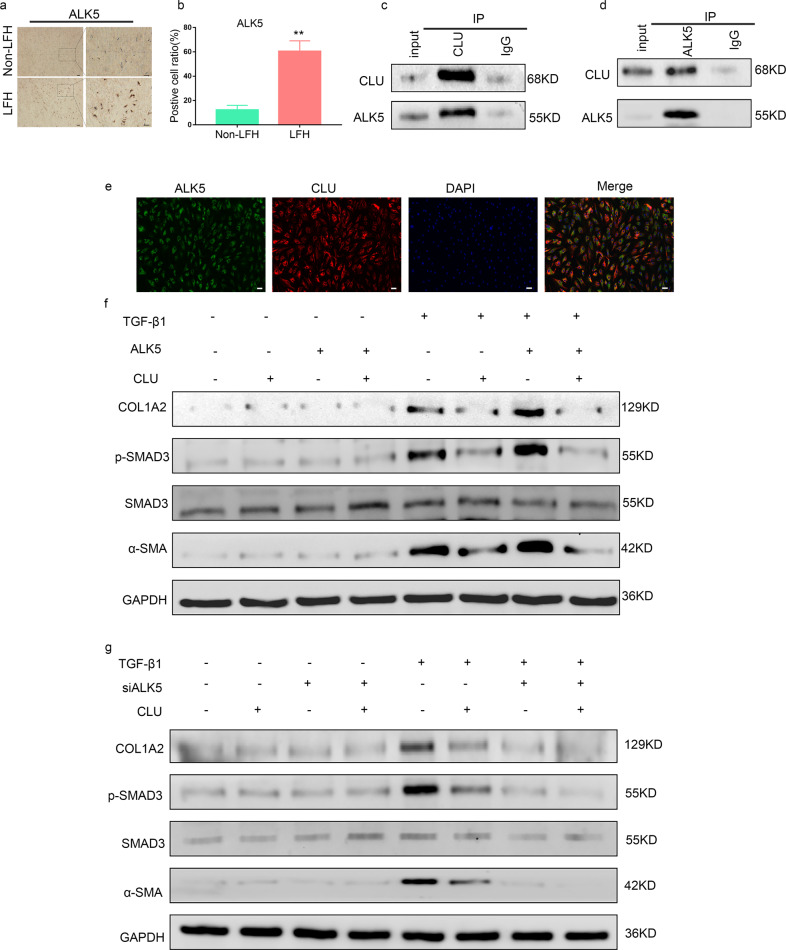
CLU inhibits TGF-β1-induced SMAD3 signaling by binding to ALK5. **a** Immunohistochemical staining of ALK5 in LF tissues from the two groups (*n* = 6). The scale bar indicates 100 μm. **b** Quantitative analysis of the positive cell ratio for ALK5. Data are shown as the mean ± S.D. **P < 0.01. **c**, **d** Reciprocal immunoprecipitation showing the interaction between ALK5 and CLU. LF cells were treated with 5 ng/ml TGF-β1 for 24 h before performing Co-IP analysis. **e** Representative images from the immunofluorescence assay showing the colocalization of ALK5 and CLU after treatment with 5 ng/ml TGF-β1 using specific antibodies. ALK5 (green), CLU (red) and DAPI (blue). The scale bar indicates 50 μm. **f** Western blot analysis showed the effects of ALK5 overexpression on the SMAD3 signaling pathway protein levels upon treatment with TGF-β1 and CLU or not. **g** Effects of siALK5 on the protein levels of the SMAD3 signaling pathway upon treatment with TGF-β1 and CLU or not.

Furthermore, the molecular mechanism underlying the ability of CLU to negatively regulate SMAD3 signaling was explored. IHC was conducted to confirm that the ratio of ALK5-positive cells was increased in LFH samples (Fig. 5a, b). Previous genome-wide screening showed that CLU interacts with ALK5^21,22^. Therefore, TGF-β1-treated LF cells were used to explore the physical interaction between endogenous ALK5 and CLU using Co-IP analysis. The results showed that the anti-CLU antibody and anti-ALK5 antibody pulled down each other (Fig. 5c, d). Moreover, the intracellular interaction was explored through immunofluorescence techniques. The results showed that the two proteins were distributed in the same region, and the composite image indicated that they were mostly colocalized (Fig. 5e).

ALK5 directly phosphorylates SMAD3, resulting in its subsequent nuclear translocation. Therefore, we hypothesized that CLU inhibited the phosphorylation of SMAD3 by ALK5 in LF cells, thus inhibiting SMAD3 signaling. ALK5 was thus overexpressed using a transfection plasmid in LF cells. The findings showed that CLU effectively inhibited the TGF-β1-induced SMAD3 signaling pathway and fibrotic marker expression (Fig. 5f). In addition, the TGF-β1-induced SMAD3 signaling pathway and the expression of fibrotic markers were suppressed through silencing ALK5 (Fig. 5g). However, CLU had no significant effect on the SMAD3 signaling pathway or fibrotic marker levels without TGF-β1 treatment (Fig. 5f, g). Prediction using the signaling model indicated that CLU inhibits TGF-β1-induced SMAD3 signaling by competitively binding to ALK5 in LF cells.

### CLU was stabilized by PRKD3 through inhibition of lysosomal degradation of CLU

**Fig. 6:**
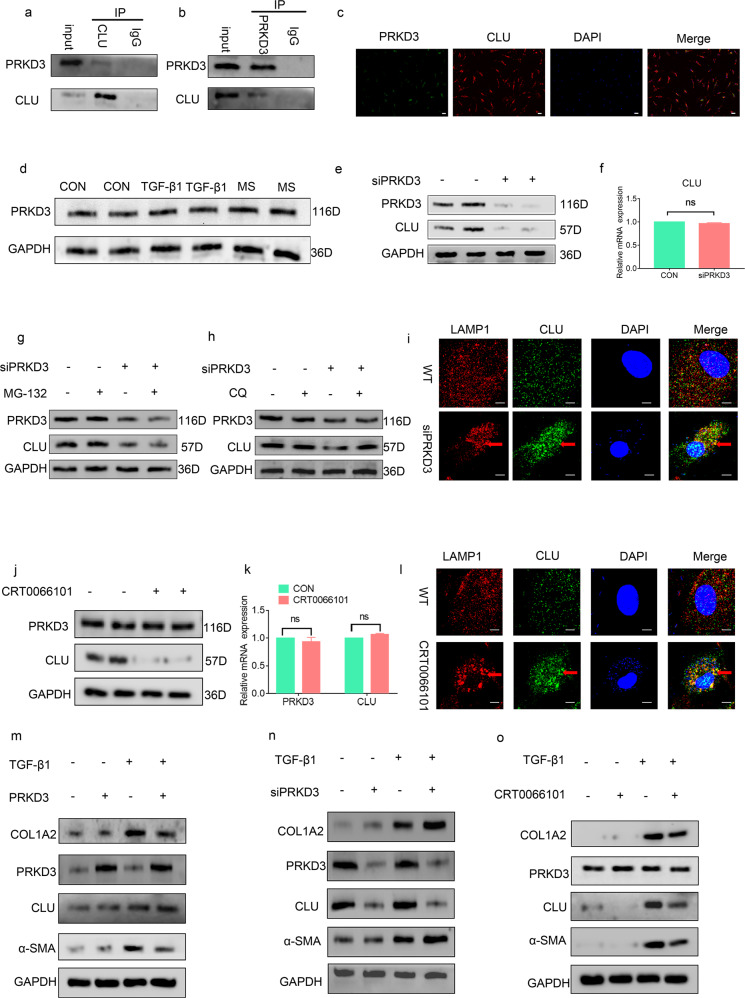
PRKD3 suppressed lysosomal CLU degradation via its kinase activity. **a, b** Reciprocal immunoprecipitation showing the interaction between PRKD3 and CLU. **c** Representative images from the immunofluorescence assay showing the colocalization of PRKD3 and CLU using specific antibodies. PRKD3 (green), CLU (red) and DAPI (blue). The scale bar indicates 50 μm. **d** Representative western blot showing that the protein level of CLU was not significantly different among the three groups. **e** Representative western blot showing that treatment of LF cells with siPRKD3 decreased CLU protein levels compared with the expression level of WT cells. **f** RT‒PCR findings showing that there were no marked differences in CLU mRNA levels in the siPRKD3 LF cells compared with the WT cells. **g**, **h** Western blots showing that treatment with the lysosomal degradation suppressor CQ but not the proteasomal degradation suppressor MG132 elevated the protein levels of CLU in the siPRKD3-treated LF cells. **i** Representative confocal images from the immunofluorescence assay showing a significant increase in CLU-LAMP1 colocalization foci in the siPRKD3-treated LF cells compared with the WT cells. Representative CLU-LAMP1 colocalization foci are shown by red arrows. Scale bar: 5 µm. **j** Representative western blots showing a decrease in CLU protein levels in LF cells after treatment with CRT0066101 compared with the cells treated with PBS. **k** RT‒PCR results showing that there was no marked difference in CLU and PRKD3 mRNA levels after treatment with the PRKD3 kinase activity inhibitor CRT0066101 relative to treatment with PBS. **l** Representative confocal images from immunofluorescence assays showing a significant increase in CLU-LAMP1 colocalization foci in LF cells upon treatment with CRT0066101 compared with treatment with PBS. Representative CLU-LAMP1 colocalization foci are shown by the red arrow. Scale bar: 5 µm. **m** Representative western blots showing the effects of overexpression of PRKD3 on the protein levels of ECM proteins in LF cells with or without TGF-β1 treatment. **n** Representative western blots showing the effects of siPRKD3 on the protein levels of ECM proteins in LF cells with or without TGF-β1 treatment. **o** Representative western blots showing the effects of CRT0066101 on the protein levels of ECM proteins in LF cells with or without TGF-β1 treatment.

Furthermore, the inhibition of CLU metabolism was explored. The association between endogenous CLU and PRKD3 was validated by immunofluorescence and Co-IP analyses (Fig. 6a, b, c). Moreover, analysis was conducted to verify that mechanical stress and TGF-β1 had no significant effect on PRKD3 compared with PBS (Fig. 6d). CLU protein and gene expression levels were determined using siPRKD3 compared with the control to further evaluate whether and how PRKD3 functions on CLU through direct binding. The results indicated that PRKD3 positively regulated the protein levels of CLU (Fig. 6e) and that PRKD3 did not regulate CLU gene transcription (Fig. 6f).

Further analysis was conducted to explore the mechanism underlying the stabilization of CLU protein by PRKD3. A proteasomal suppressor (MG132) and lysosomal suppressor (chloroquine, (CQ)) were used to explore whether PRKD3 stabilizes the CLU protein via the ubiquitin-proteasome system (UPS)^21^ or autophagy-lysosome pathway (ALP)^23^ or both. The results showed that CQ but not MG132 alleviated CLU degradation upon siPRKD3 treatment (Fig. 6g, h). LAMP1^24^ antibodies were used to label lysosomes to further explore whether PRKD3 suppresses lysosomal CLU degradation by decreasing CLU transportation to lysosomes. Immunofluorescence assays showed that siPRKD3 increased CLU distribution in lysosomes relative to the controls (Fig. 6i). Moreover, the pan-PRKD inhibitor CRT0066101^25^ was used to determine whether PRKD3 suppresses lysosomal CLU degradation through its kinase activity. The results showed that inhibition of PRKD3 kinase activities by CRT0066101 reduced CLU protein levels but not CLU mRNA levels (Fig. 6j, k). Furthermore, CRT0066101 treatment increased CLU lysosomal distribution relative to the WT (Fig. 6l).

Further analysis was conducted to explore the function of CLU stabilization by PRKD3 in LF cells. A PRKD3 overexpression assay was performed, and the results consistently showed that overexpression of PRKD3 in LF cells increased CLU protein levels and reduced ECM protein levels upon TGF-β1 treatment (Fig. 6m). Furthermore, a siPRKD3 assay was conducted, and the findings showed significantly reduced CLU protein levels and increased ECM protein levels upon TGF-β1 treatment (Fig. 6n). Moreover, a CRT0066101 assay was performed to explore whether CRT0066101 downregulated CLU protein expression and increased ECM protein expression upon TGF-β1 treatment (Fig. 6o). The findings indicate that in vitro CLU was stabilized by PRKD3 through the inhibition of lysosomal degradation of CLU.

### CLU prevented mechanical stress-induced LFH in vivo

**Fig. 7:**
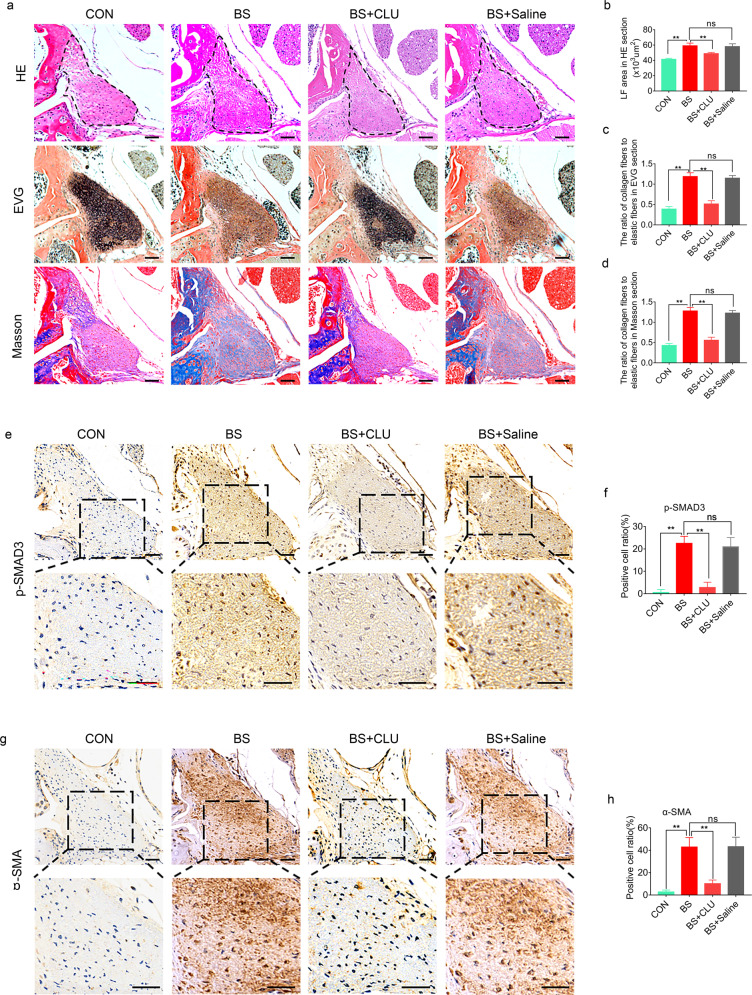
CLU ameliorates mechanical stress-induced LFH in vivo. **a** Representative images of H&E, EVG, and Masson staining of tissues from mice under different treatments for 8 weeks. The scale bar indicates 50 μm. **b**–**d** Quantitative analysis of the LF area and the ratio of collagen fibers to elastic fibers in EVG and Masson-stained sections (*n* = 6 mice in each group). Data are expressed as the mean ± S.D. **P < 0.01. ns, not significant. **e**, **g** Representative images of immunohistochemical staining for p-SMAD3 and α-SMA in mouse tissues under different treatments after 8 weeks. The scale bar indicates 50 μm. **f**, **h** Quantitative analysis of the ratio of p-SMAD3-positive cells and α-SMA-positive cells (*n* = 6 mice in each group). Data are expressed as the mean ± S.D. **P < 0.01. ns, not significant.

The above results indicate that CLU inhibits TGF-β1-induced SMAD3 signaling through competitive binding of ALK5 in vitro and that CLU is stabilized by PRKD3, thus inhibiting fibrosis. Further experiments were conducted to explore whether CLU prevents mechanical stress-induced LFH in vivo. The results showed that the average LF area in the 8-week bipedal standing mice markedly increased relative to that in the control group (Fig. 7a, b). Moreover, the elastic fiber to collagen fiber ratio decreased in the bipedal standing group compared with the control (Fig. 7a, c, d). These results indicate that the model was successfully established. The bipedal standing group was intravenously administered CLU or saline to further explore the role of CLU in mechanical stress-induced LFH. H&E staining revealed that CLU reduced the LF area in the bipedal standing mice (Fig. 7a, b). Moreover, CLU increased the elastic fiber to collagen fiber ratio (Fig. 7a, c, d). Immunohistochemical staining analysis showed that CLU decreased the mechanical stress-induced increase in the ratio of p-SMAD3-positive cells (Fig. 7e, f). Furthermore, CLU decreased the mechanical stress-induced ratio of α-SMA-positive cells in vivo (Fig. 7g, h). However, the difference between the bipedal standing group and the saline + bipedal standing group was not statistically significant.

### CLU and PRKD3 were stable in peripheral blood

**Fig. 8:**
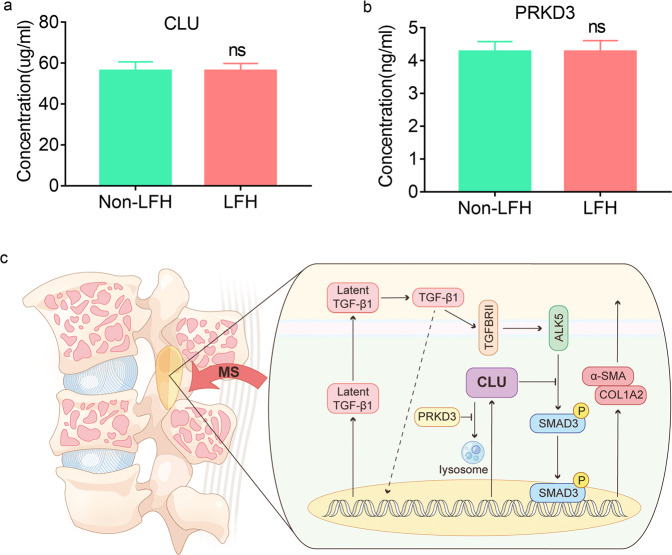
CLU and PRKD3 expressions in peripheral blood and a proposed working model. **a**, **b** ELISA analysis of CLU and PRKD3 protein levels in serum samples from LFH patients (*n* = 17) and non-LFH patients (*n* = 25). Data are expressed as the mean ± S.D. ns, not significant. **c** Mechanical stress increased CLU expression as well as TGF-β1, and TGF-β1 treatment also increased CLU expression as well as α-SMA and COL1A2. Further results revealed that CLU inhibited mechanical stress-stimulated and TGF-β1-induced SMAD3 activities through suppression of SMAD3 phosphorylation. The results also revealed that CLU inhibits phosphorylation and/or nuclear translocation by competitively binding to ALK5. In addition, they demonstrated that PRKD3 stabilizes CLU protein by inhibiting lysosomal distribution and degradation of CLU.

The results showed no significant difference in serum CLU concentrations from blood samples between the LFH group and the non-LFH group (Fig. 8a); however, CLU was highly expressed in LFH samples (Fig. 1a, b). Similarly, the PRKD3 expression level was not significantly different between the two groups (Fig. 8b).

## Discussion

Previous studies have shown that LFH plays a key role in the pathogenesis of LSCS^5,26^. Although several studies have investigated the mechanisms of LFH at the cellular and molecular levels, the precise pathomechanism has not been fully elucidated^2,27,28^. In the current study, a novel bipedal standing mouse model was established^29^. The animal model is more suitable for understanding the physiological process of LFH caused by mechanical stress than other models. In our previous study, spinal degeneration between bipedal standing mice and aged quadruped standing mice was compared. The findings showed that age had a significant effect on the spinal anterior column, and mechanical stress significantly affected the spinal posterior column^29^. The results also demonstrated that the area of LF in bipedal mice was significantly larger than that in 18-month-old mice. The findings indirectly indicated that mechanical stress acts on the LF with advances in age because humans are bipedal standing animals. Clinical research has shown that heavy manual workers are more prone to LSCS than the general population^30^. This finding implied that mechanical stress is the main initiating factor of fibrosis and LFH.

ECM remodeling is a critical component of LF fibrosis and LFH. Excess deposition of the ECM leads to LF fibrosis or LF scarring. TGF-β1 has a fundamental role in the stimulation of ECM production mediated by mechanical stress; therefore, appropriate resolution of tissue repair is critical to prevent adverse effects. CLU was identified as a factor that is initiated by mechanical stress and TGF-β1. In our study, CLU levels were significantly increased in LFH specimens and elevated relative to lower expression levels of target genes for fibrosis. The results indicate that the increase in CLU that occurs during LF remodeling inhibits the progression of LF hyperplasia. This result was verified by injection of exogenous CLU in the bipedal standing mouse models, which caused a significant reduction in LFH. Large human sample sizes and CLU knockout animals should be used to establish the association between CLU levels and LFH in further research.

The in vitro study showed that CLU inhibits the phosphorylation of SMAD3 by modulating TGF-β1 signaling at the SMAD3 pathway. Therefore, low levels of p-SMAD3 are translocated to the nucleus, ultimately suppressing TGF-β1 target genes^12^. Gwon-Soo^11^ reported that CLU acts downstream of the TGF-β1 receptor, implying that CLU is a weak sensor of TGF-β1 signaling levels at higher concentrations. Consequently, TGF-β1 signaling attains sufficient strength and duration to induce CLU, and CLU induces negative feedback, thus inhibiting ECM production through inhibition of SMAD3 signaling as well as downregulation of target gene levels.

Mechanistic studies have shown that CLU suppresses TGF-β1 signaling by directly and competitively interacting with ALK5^18,21^. ALK5 is a TGF-β1 type I receptor and a transmembrane Ser/Thr kinase receptor. ALK5 binds to TGF-β1 type II serine/threonine kinase receptor to induce transmembrane serine/threonine kinase^31^. ALK5 transduces TGF-β1 signaling from cell surfaces to the cytoplasm, thus modulating physiological and pathological processes, such as cell cycle arrest, mesenchymal cell proliferation and differentiation, wound healing, ECM production, immunosuppression and carcinogen expression^32^. Sunghyeok^33^ reported that the activity of the TGF‐β1 pathway was inhibited in ALK5 KO LF cells and that the expression of TGF-β1 target genes was reduced. Furthermore, in the present study, ALK5 overexpression and siALK5 studies showed that CLU inhibited TGF-β1/SMAD3 signaling by competitively binding to ALK5^18^. These findings indicate that CLU inhibits TGF-β1/ALK5-mediated SMAD3 phosphorylation, thus inhibiting the nuclear localization of p-SMAD3. Notably, CLU upregulation failed to inhibit LF hypertrophy, indicating that there are other signaling pathways, such as mTORC1 signaling^34^ and Wnt/β-catenin signaling, involved in the regulation of LFH and fibrosis^35^.

The results of the current study indicated that CLU synthesis increased under mechanical stress and TGF-β1 conditions. Moreover, the mechanism underlying the inhibition of CLU metabolism was explored. PRKD3 is a key regulator that protects mice against liver fibrosis^36^. In addition, PRKD3 promotes triple-negative breast cancer tumor growth by inhibiting the lysosomal distribution and degradation of CLU^37^. In the present study, the findings showed that PRKD3 stabilizes CLU to prevent decomposition (Fig. 8c). As a key regulator of CLU, PRKD3 is involved in the mechanisms of CLU in LFH pathogenesis. This finding provides a new promising approach for designing targeted therapy to inhibit CLU catabolism by targeting PRKD3 for effective management of LFH.

In the present study, ELISAs were conducted on human LF samples and blood samples between the LFH group and the non-LFH group. The results showed significantly increased expression of CLU in LF samples; however, there was no marked difference in CLU levels in blood samples from the LFH group and non-LFH group. CLU upregulation has been reported in local tissue, such as OA-induced cartilage degeneration^38^, osteoporotic disease^39^, tears of dry eye^19^ and brain tissues of AD patients. The results suggested specifically high expression of CLU in LF tissues prone to fibrosis. This tissue specificity prevented CLU from serving as an early diagnostic indicator of LSCS, but LF specifically expressing CLU suggests fewer systemic side effects of the potential corresponding treatment and therefore has high clinical significance.

Transdifferentiation of LF cells from fibroblasts to myofibroblast-like cells under mechanical stress and TGF-β1 induction is the main source of ECM. Progressive fibrosis leads to LFH, and surgery is currently the only treatment option for end-stage LFH. Therefore, further elucidating the role of CLU in LFH induced by mechanical stress through TGF-β1 signaling has clinical significance in the alleviation of LSCS.

Nevertheless, this study has some limitations. The iTRAQ data were only from three pairs of patient samples. Moreover, our study did not evaluate LF cell subpopulations or the CLU positioning of these LF cell subpopulations. Single-cell RNA-sequencing studies may help elucidate this issue.

## Supplementary information

Supplementary Table

## Data Availability

The data for this study are available by contacting the corresponding authors, Zhongmin Zhang and Liang Wang, upon reasonable request.
