# Supplementary material for: Clusterin negatively modulates mechanical stress-mediated ligamentum flavum hypertrophy through TGF-β1 signaling
Source: Exp Mol Med. 2022 Sep 21;54(9):1549–62. doi: 10.1038/s12276-022-00849-2 (PMC9534863; doi:10.1038/s12276-022-00849-2)
Supplement: Supplementary file 1 — Supplementary Table [file 12276_2022_849_MOESM1_ESM.pdf]

Supplementary Table 1. The Sequencing Primers

Genes	Primer sequences
siALK5	F: 5’-GACUUAAUUCCUCGAGAUATT-3’
	R: 5’- UAUCUCGAGGAAUUAAGUCTT-3’
siPRKD3	F: 5’- CGAUAUGUCAGUACUGCAATT-3’
	R: 5’- UUGCAGUACUGACAUAUCGTT-3’

Supplementary Table 2. The qRT-PCR primer sequence

Genes	Primer sequences
CLU	F: 5’- AGCTCGCTGAGAAGTTCACC-3’
	R: 5’- TTGTGGGAAGACACCGTGG-3’
TGF-β1	F: 5’-TACCTGAACCCGTGTTGCTC-3’
	R: 5’- CCGGTAGTGAACCCGTTGAT-3’
COL1A2	F: 5’-TGGTCTCGGTGGGAACTTTG-3’
	R: 5’- CACCCTGTGGTCCAACAACT-3’
α-SMA	F: 5’- AGCCAAGCACTGTCAGGAAT-3’
	R: 5’- CACCATCACCCCCTGATGTC-3’
PRKD3	F: 5’- GAGCCTGCCACTGCTAACTA-3’
	R: 5’- GTCCTCATTTTCATTCTGGGGG-3’
GAPDH	F: 5’- GGATTTGGTCGTATTGGGCG-3’
	R: 5’- TCCCGTTCTCAGCCATGTAGT-3’

Supplementary Table 3.

Variable	Non-LFH Group	LFH Group	P value
	(n=22)	(n=22)	
Age(years)	44.91 ± 2.59	61.23 ± 1.28	<0.01
Gender(male:female)	12:10	12:12	
LF thickness	3.05 ± 0.09	5.91 ± 0.20	<0.01
Lumbar level	L4/5	L4/5	
